# Unveiling the potential of diffusion model-based framework with transformer for hyperspectral image classification

**DOI:** 10.1038/s41598-024-58125-4

**Published:** 2024-04-10

**Authors:** Neetu Sigger, Quoc-Tuan Vien, Sinh Van Nguyen, Gianluca Tozzi, Tuan Thanh Nguyen

**Affiliations:** 1 School of Computing, The University of Buckingham, Buckingham, MK181EG UK; 2 Faculty of Science and Technology, Middlesex University, London, UK; 3 School of Computer Science and Engineering, International University-Vietnam National University of HCMC, Ho Chi Minh City, Vietnam; 4 School of Engineering, University of Greenwich, Chatham Maritime, ME44TB UK; 5 School of Computing and Mathematical Sciences, University of Greenwich, London, SE109LS UK

**Keywords:** Computational science, Computer science

## Abstract

Hyperspectral imaging has gained popularity for analysing remotely sensed images in various fields such as agriculture and medical. However, existing models face challenges in dealing with the complex relationships and characteristics of spectral–spatial data due to the multi-band nature and data redundancy of hyperspectral data. To address this limitation, we propose a novel approach called DiffSpectralNet, which combines diffusion and transformer techniques. The diffusion method is able extract diverse and meaningful spectral–spatial features, leading to improvement in HSI classification. Our approach involves training an unsupervised learning framework based on the diffusion model to extract high-level and low-level spectral–spatial features, followed by the extraction of intermediate hierarchical features from different timestamps for classification using a pre-trained denoising U-Net. Finally, we employ a supervised transformer-based classifier to perform the HSI classification. We conduct comprehensive experiments on three publicly available datasets to validate our approach. The results demonstrate that our framework significantly outperforms existing approaches, achieving state-of-the-art performance. The stability and reliability of our approach are demonstrated across various classes in all datasets.

## Introduction

Hyperspectral images (HSI) are now being captured more effectively by imaging spectrometers aboard satellites and aircraft. Unlike regular optical images with just three channels, Red, Green, Blue, each pixel of HSI contains abundant and continuous spectral information. This allows for the identification of complicated spectral characteristics of subjects that might be unnoticed. HSI is extensively used in various earth remote sensing applications, including land use and land cover classification^1^, precision agriculture^2,3^, object detection^4^, tree species classification^5^, brain cancer detection^6^, and more.

The challenges of classification in HSI arise from their high dimensionality, strong correlations between adjacent bands, a nonlinear data structure, and limited training samples^7^. To address these challenges and improve classification accuracy, researchers have proposed several methods^8^. While traditional approaches like Maximum Likelihood Classification have been foundational, they often face challenges with high-dimensional data spaces, known as the curse of dimensionality^9^. Initially, spectral information for each pixel was fed into neural networks to identify the corresponding class^10^. As data dimensionality increased, feature selection and dimensionality reduction became crucial. Techniques like principal component analysis (PCA)^11^ and support vector machine (SVM)^12^ were often employed to achieve better classification results. However, traditional techniques faced difficulties in effectively utilising the spatial–spectral relationships and capturing complex information in HSI. By considering the neighbouring pixels along with their corresponding spectral values, we can gain valuable insights into their underlying structures and extract meaningful information of different materials which ultimately enhance accurate analysis.

Convolutional neural networks (CNNs)^13,14^ have better feature representation and high accuracy in classification and have demonstrated promising performance in HSI classification. The CNNs can automatically extract hierarchical features from HSI^15^. As datasets become larger, deeper architectures like residual networks (ResNets)^16^ were introduced, specifically adapted to capture complex patterns in HSI data for classification^17^. Advanced architectures such as autoencoders were later developed to extract a compressed representation of HSI data for classification purposes^18^. Attention mechanisms were integrated into CNN architectures to enhance the accuracy of classification by weighing the importance of different spectral bands^19^. Furthermore, advancements in the CNNs led to the introduction of novel pooling and unpooling mechanisms that better preserve spatial information during classification^20^. In recent years, the CNNs have been shown to be effective in HSI classification; however, there are still several limitations. For instance, the convolutional operations handle a local neighborhood. Hence, the number of layers and kernel size restrict the CNNs’ receptive field, making it less effective at capturing long-range dependencies in input data^21^. As a result, learning the long-range dependencies of the HSI, often consisting of hundreds of spectral bands, is challenging.

Recurrent neural network (RNNs)^22^ are capable of capturing the spatial–spectral relationship from long-range sequence data, they face challenges such as vanishing gradients and dependency on the order of spectral bands. Transformers^23^, originally designed for natural language processing (NLP), have shown promising results when integrated into HSI classification. They effectively capture long-range dependencies in hyperspectral data^24,25^. Here, CNN is a vector-based method that considers the inputs as collection of pixel vectors^26^, and thus it can lead to information loss when processing with hyperspectral pixel vectors^27^. In the work^28^, a multispectral image classification framework was introduced to overcome the limitations of the CNNs in pixel-wise remote sensing classification and spectral sequence representation and, integrates fully connected (FC) layers, CNNs, and transformers. Unlike the classic transformers that focus on band-wise representations, SpectralFormer^24^ is an example of such a framework that captures spectrally local sequence information, creates group-wise spectral embeddings, and introduces cross-layer skip connections to retain crucial information across layers through adaptive residual fusion. Another novel model, namely SS1DSwin^29^, is based on transformers and implements the network architecture of swin transformer^30^. It was shown to effectively capture reliable spatial and spectral dependencies for HSI classification.

Effectively learning rich representations and addressing the complexities of spectral–spatial relations in high-dimensional data are crucial for achieving optimal HSI classification results. However, transformer-based methods face challenges in directly capturing reliable and informative spatial–spectral representations available in HSI. They generally do not fully leverage spatial information^31^ and have limitations in extracting fine-grained local feature patterns^32^. Recently, the denoising diffusion probabilistic model (DDPM)^33^ has emerged as a groundbreaking class of generative models, adept at modeling complex relationships and effectively learning high-level and low-level visual features. SpectralDiff^34^ leveraged a diffusion model to extract potent features. However, it employed a pixel-wise classification approach, which limits the ability to effectively capture and identify distinct spatial–spectral relationships in HSI.

To overcome these challenges, we have thoroughly re-evaluated the process of extracting features of the HSI data from different perspectives. Consequently, we have developed a novel HSI classification method that incorporates diffusion and transformer techniques leveraging their respective advantages. The features’ representation learned from the diffusion models have been demonstrated to be highly effective in various discriminating tasks with impressive performance like semantic segmentation^35^, object detection^36^, and face generation^37^.

This paper presents a novel classification framework called DiffSpectralNet, combining a diffusion-based spectral–spatial network with transformers. This diffusion model, a type of generative models, excels in capturing the relationships between spectral and spatial information in HSI data. Deep features are extracted both effectively and efficiently to make the most of the spectral–spatial information present in the data. The main stages of the framework are summarized as follows: first, we ultilise forward and reverse diffusion processes to learn high-level and low-level features from HSI. Second, to make effective use of the extensive timestamps-wise features, we extract intermediate hierarchical features from the denoising U-Net at different timestamps. Subsequently, we employ a proposed supervised transformer-based classifier for performing HSI classification.

We examine the effectiveness of the proposed method conducted on three widely known datasets that their download link can be found in the Data availability section. Our results clearly demonstrate that the proposed method significantly improves classification results and outperforms other advanced HSI classification methods. Moreover, this study also opens the way for further investigations into the potential of diffusion models in learning high- and low-level spectral–spatial features with significant flexibility in HSI. Ongoing research will likely enhance the application of diffusion models in processing complex, high-dimensional hyperspectral data, opening up promising prospects for diverse applications.

## Results

In this section, we begin by providing an introduction to three different experimental datasets for HSI. After that, we delve into the details of the experimental results that have been produced by our proposed model. In addition, we conduct a thorough analysis of parameters of the framework to gain a better understanding of their significance and implications.

**Table 1:** Details of Indian Pines, Pavia University, and Salinas Scene Datasets.

Indian Pines Dataset		Pavia University Dataset		Salinas Scene Dataset	
Land cover type	Samples	Land cover type	Samples	Land cover type	Samples
Alfalfa	46	Asphalt	6631	Brocoli_green_weeds_1	2009
Corn-notill	1428	Meadows	18649	Brocoli_green_weeds_2	3726
Corn-min	830	Gravel	2099	Fallow	1976
Corn	237	Trees	3064	Fallow_rough_plow	1394
Grass/pasture	483	Painted metal sheets	1345	Fallow_smooth	2678
Grass/trees	730	Bare Soil	5029	Stubble	3959
Grass/pasture-mowed	28	Bitumen	1330	Celery	3579
Hay-windrowed	478	Self-Blocking Bricks	3682	Grapes_untrained	11,271
Oats	20	Shadows	947	Soil_vinyard_develop	6203
Soybeans-notill	972			Corn_senesced_green_weeds	3278
Soybeans-min	2455			Lettuce_romaine_4wk	1068
Soybeans-clean	693			Lettuce_romaine_5wk	1927
Wheat	205			Lettuce_romaine_6wk	916
Woods	1265			Lettuce_romaine_7wk	1070
Bldg-grass-tree-drives	386			Vinyard_untrained	7268
Stone-steel towers	93			Vinyard_vertical_trellis	1807
Total	10,349	Total	42,776	Total	54,129

### Dataset

Three well-known available datasets, Indian Pines, Pavia University and Salinas Scene, were used to examine the classification performance. Number of categories and their correspondent samples were shown in Table 1. First, the Indian Pines dataset collected in 1992 using the Airborne Visible Infrared Imaging Spectrometer (AVIRIS) Sensor, covering the northwestern region of Indiana in the United States. It consists of $145 \times 145$ pixels with each pixel having a spatial resolution of 20 metres (m) and 220 spectral bands in the wavelength range of 400–2500 nm. The dataset contains labeled pixels with 16 categories. We use 10% of the labeled samples for training and the rest for testing. The second HSI dataset, Pavia University, was acquired by the Reflective Optics System Imaging Spectrometer (ROSIS) sensor. The ROSIS sensor acquired 103 bands covering the spectral range from 430 to 860 nm, and the dataset consists of $610 \times 340$ pixels at GSD of 1.3 m. Moreover, there are 9 land cover classes in the dataset. We use $5\%$ of the labeled samples for training and the rest for testing. Lastly, Salinas Scene dataset was collected using the AVIRIS sensor and is situated in Salinas Valley, California. The spatial resolution is set at 3.7 m. and the dataset includes 16 crop types and has been widely utilized in classification. After the exclusion of 20 bands associated with water vapor and noise, a total of 204 bands remained, resulting in a data size of $512 \times 217$. We use $5\%$ of the labeled samples for training and the rest for testing.

### Training process

We used the PyTorch framework to implement and train the DiffSpectralNet model. The training was done on a basic hardware setup, which consists of a POWER8NVL production-grade CPU with 128 CPU threads spread across 2 sockets for efficient processing. Additionally, four NVIDIA Tesla P100 GPUs were used for enhanced graphical computations, each offering a memory of approximately 16 GB.

The diffusion model was optimised using the Adam optimizer and trained for 30, 000 epochs for all datasets. We set the learning rate to $1 \times 10^{-4}$, with a batch size of 128 and a patch size of $32 \times 32$. Due to hardware limitations, we use batch size 64 for the Salinas scene dataset. To determine the amount of spectral information preserved in the compressed data, we employed PCA. Given that each dataset presents a distinct number of features post-pre-training with the diffusion model, the range of PCA components varies among three datasets. The classification model was trained using the Adam optimizer, maintaining the same learning rate of $1 \times 10^{-4}$ and a batch size of 128 for Indian Pines, Pavia University, and 64 for Salinas Scene. The size of feature patch is empirically set as 7 $\times$ 7. The number of epochs was set to 300 for Indian Pines and 600 for Pavia University and Salinas Scene datasets.

### Performance evaluation

We evaluate the performance using three prominent metrics: overall accuracy (OA), average accuracy (AA), and Kappa coefficient ($\kappa$). OA gives a direct insight into general model performance, and AA ensures each class has a balanced contribution, especially in imbalanced datasets. On the other hand, $\kappa$ measures the reliability between the ground truth and model predictions.

**Table 2:** Classification results of different HSIs, and the best result is bolded.

Model	Indian Pines			Pavia University			Salinas Scene		
	OA (%)	AA (%)	$\kappa$	OA (%)	AA (%)	$\kappa$	OA (%)	AA (%)	$\kappa$
DMVL + SVM	78.01	84.98	0.7531	86.96	80.10	0.8246	94.60	94.59	0.9400
3DCAE	92.35	92.04	–	95.39	95.36	-	95.81	97.45	–
GSSCRC	91.33	93.81	0.9013	95.77	94.13	0.9438	95.62	97.30	0.9384
SS1DSwin	89.66	94.13	0.8819	93.04	91.92	0.9068	95.45	97.78	0.9493
SpectralFormer	81.76	87.81	0.7919	91.07	90.20	0.8805	96.27	97.82	0.9585
SpectralDiff	93.15	96.43	0.9217	94.77	93.84	0.9306	98.97	99.46	0.9885
Ours	**99.06**	**98.00**	**0.9893**	**99.74**	**99.18**	**0.9965**	**99.87**	**99.82**	**0.9986**

To demonstrate the effectiveness of our proposed DiffSpectralNet, we compare its classification performance with various state-of-the-art approaches, and the following methods were chosen: DMVL^38^, similar to our proposed model, follows the two-stage algorithms. It performs unsupervised feature extraction followed by classification using an SVM classifier. 3DCAE^39^ is an unsupervised method to learn spectral–spatial features. It uses the encoder–decoder backbone with 3D convolution operations, GSSCRC^40^ algorithm incorporates the cooperative representation classification model and introduces the geodesic distance calculation method to select spectral nearest-neighbour information, thereby effectively utilising the neighbour information in HSI. This approach facilitates the exploration and utilization of the spatial–spectral neighbourhood structure of HSI data for classification. SS1DSwin^29^ design reveals local and hierarchical spatial–spectral links through two modules: the Groupwise Feature Tokenization Module (GFTM) and the 1DSwin Transformer with Cross-Block Normalized Connection Module (TCNCM). GFTM processes overlapping cubes and uses multihead self-attention for spatial–spectral relationships. Meanwhile, TCNCM utilises window-based strategies for spectral relationships and cross-block feature fusion. SpectralFormer^24^ uses transformers from a sequential perspective for classification, learns spectrally local sequence information from neighbouring bands of HSI, yielding group-wise spectral embeddings. Also, to reduce the possibility of losing valuable information in the layer-wise propagation process, a cross-layer skip connection is devised from shallow to deep layers by adaptively learning across layers. We conducted experiments on the Salinas dataset, not covered in the SpectralFormer^24^, using the same train-test split ratio employed in our experiments for consistent comparison. SpectralDiff^34^ employs an unsupervised feature extraction using a spectral–spatial diffusion module. These features are then processed per pixel by the supervised attention-based classification module.

It is worth mentioning that we directly used the outcomes reported in the papers of each of these methods. Both CNN-based and transformer-based methods produced good classification results.

**Figure 1:**
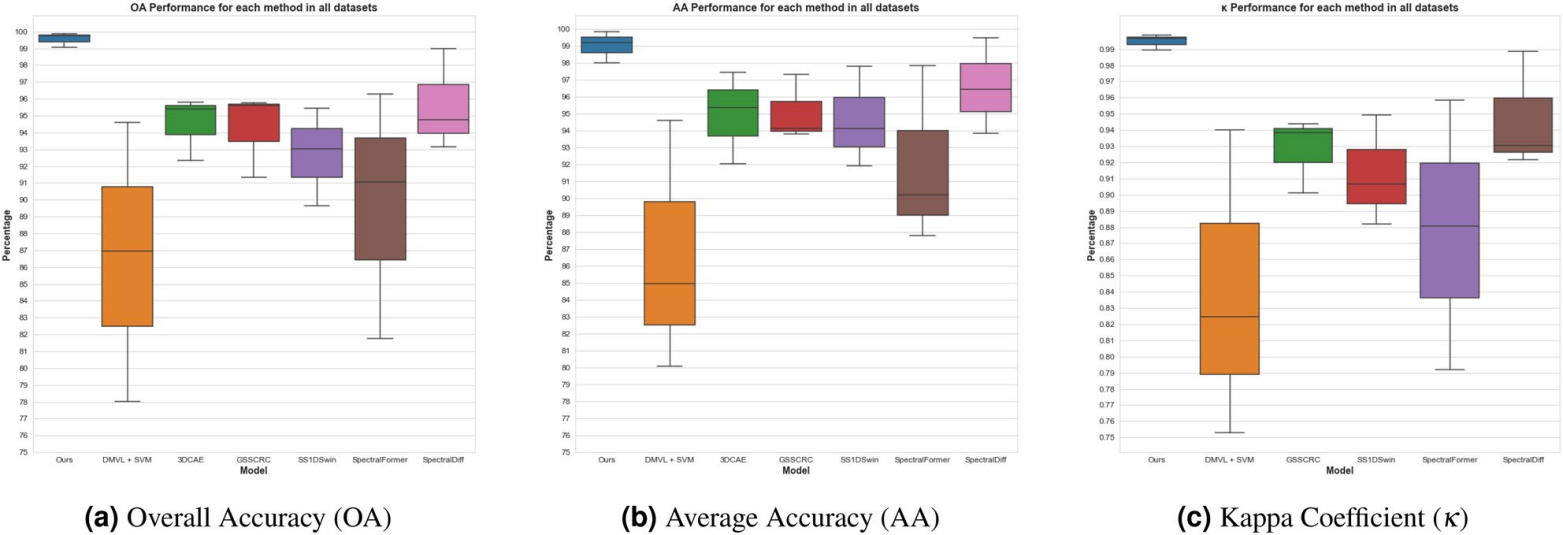
The boxplots to visualise the performance of each model using three prominent metrics. Note that, there is no published result of 3DCAE on these datasets, therefore it was not included in the visualisation.

Based on the analysis of classification results obtained for the Indian Pines, Pavia University, and Salinas Scene datasets presented in Table 2, the DiffSpectralNet algorithm proposed in this study shows improved classification accuracy for most ground objects when compared to other classification methods. The proposed method achieves the best OA, AA, and $\kappa$ values, with OA reaching $99.06\%, 99.74\, \%, \text {and}\ 99.87\%$ on the Indian Pines, Pavia University and Salinas Scene datasets, respectively. Visualisation in the Fig. 1 clearly shows DiffSpectralNet outperformed others. Moreover, we conducted additional statistical analyses using Analysis of Variance (ANOVA) and Mann-Whitney U Test, both with a confidence score of $95\%$. The *p value* from these tests were lower than 0.05, indicating that DiffSpectralNet’s performance is significantly different across three measurement metrics. All of these results proves that the DiffSpectralNet algorithm efficiently and effectively learns low and high-level features using the diffusion model. Additionally, the DiffSpectralNet algorithm leverages the combination of spectral and spatial information, enabling it to extract a greater amount of information for classification. Therefore, the DiffSpectralNet algorithm proposed in this study demonstrates promising potential for improving the accurate classification of ground objects.

**Figure 2:**
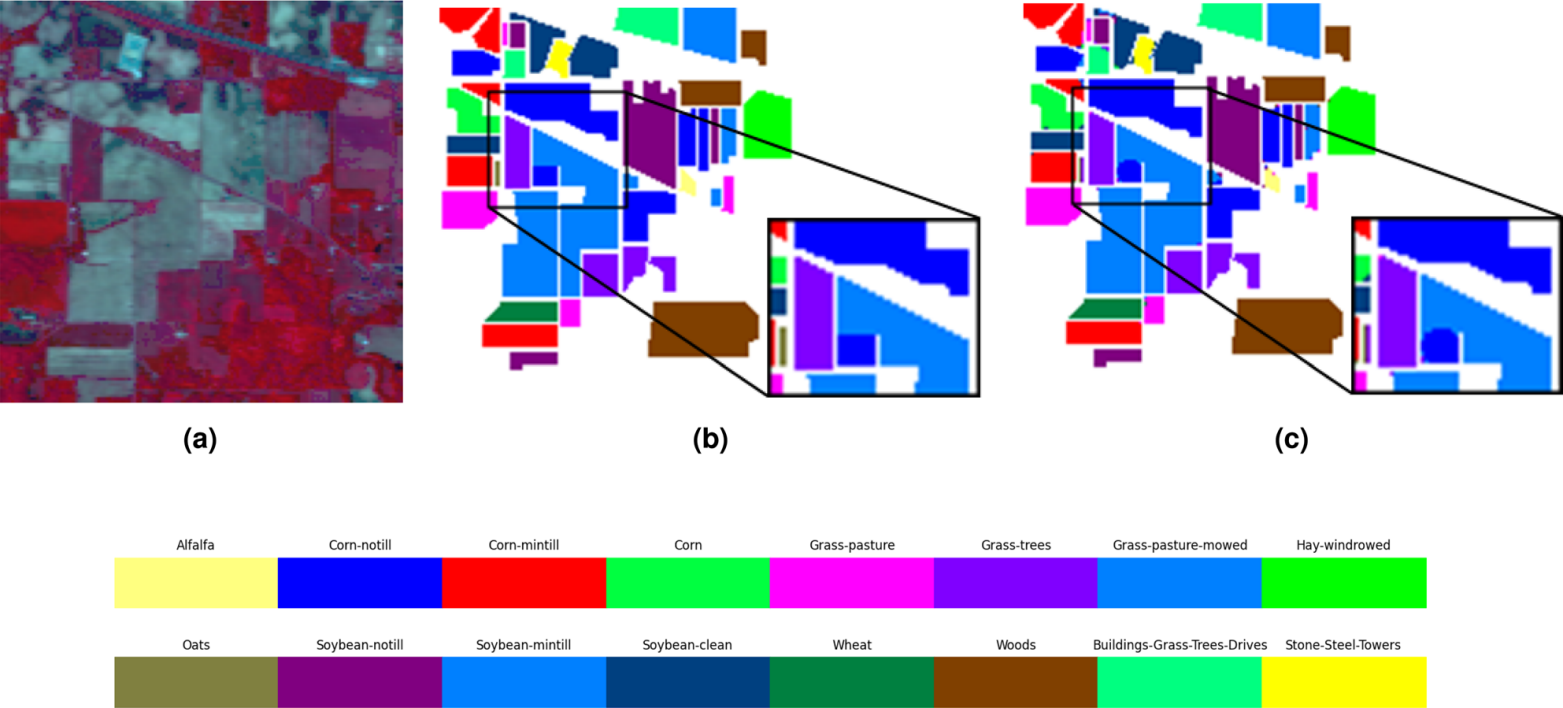
Classification results of on the Indian Pines dataset (**a**) Original HSI (**b**) ground truth (**c**) proposed method.

**Figure 3:**
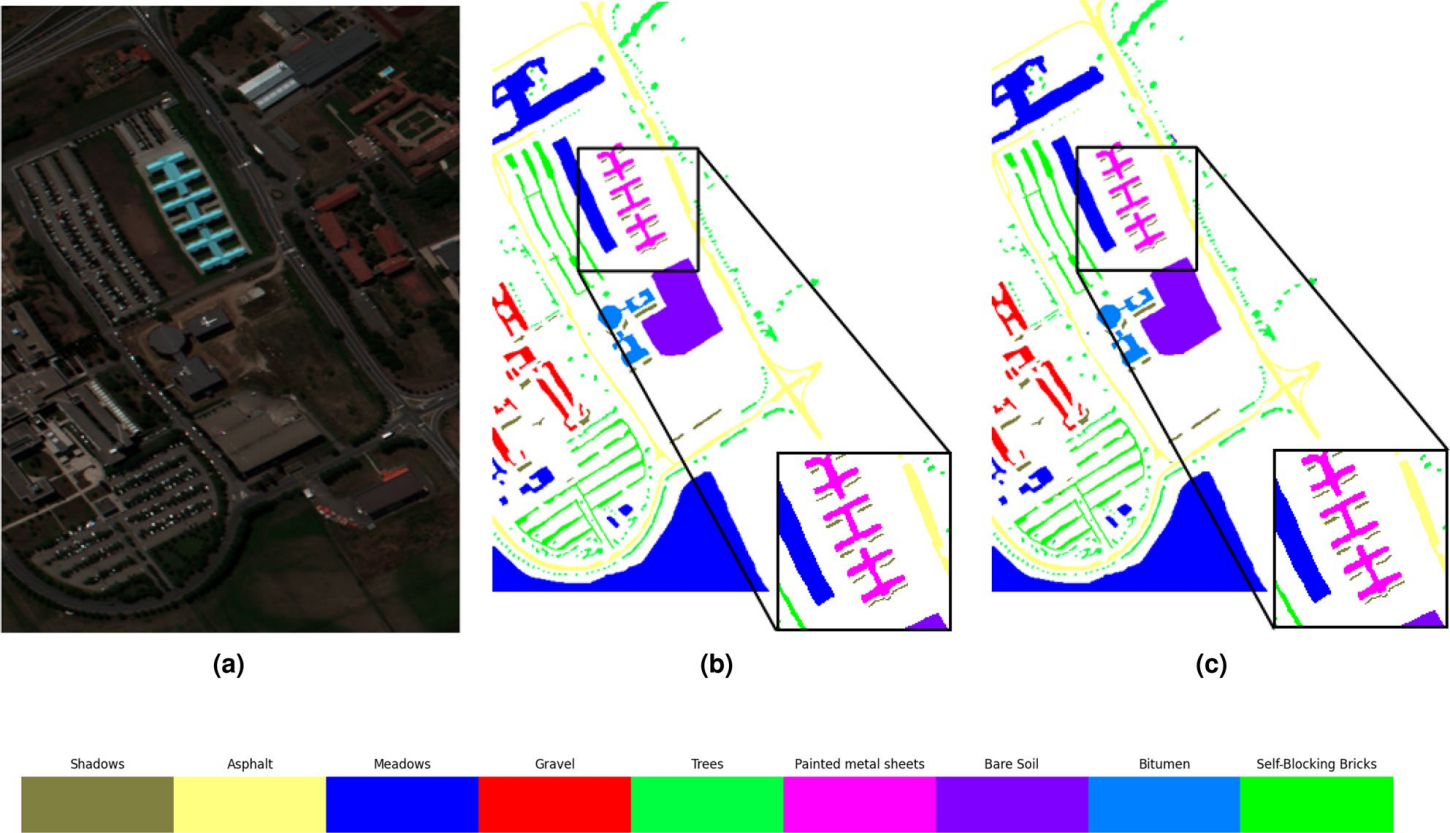
Classification results of on the Pavia University dataset (**a**) original HSI (**b**) ground truth (**c**) proposed method.

**Figure 4:**
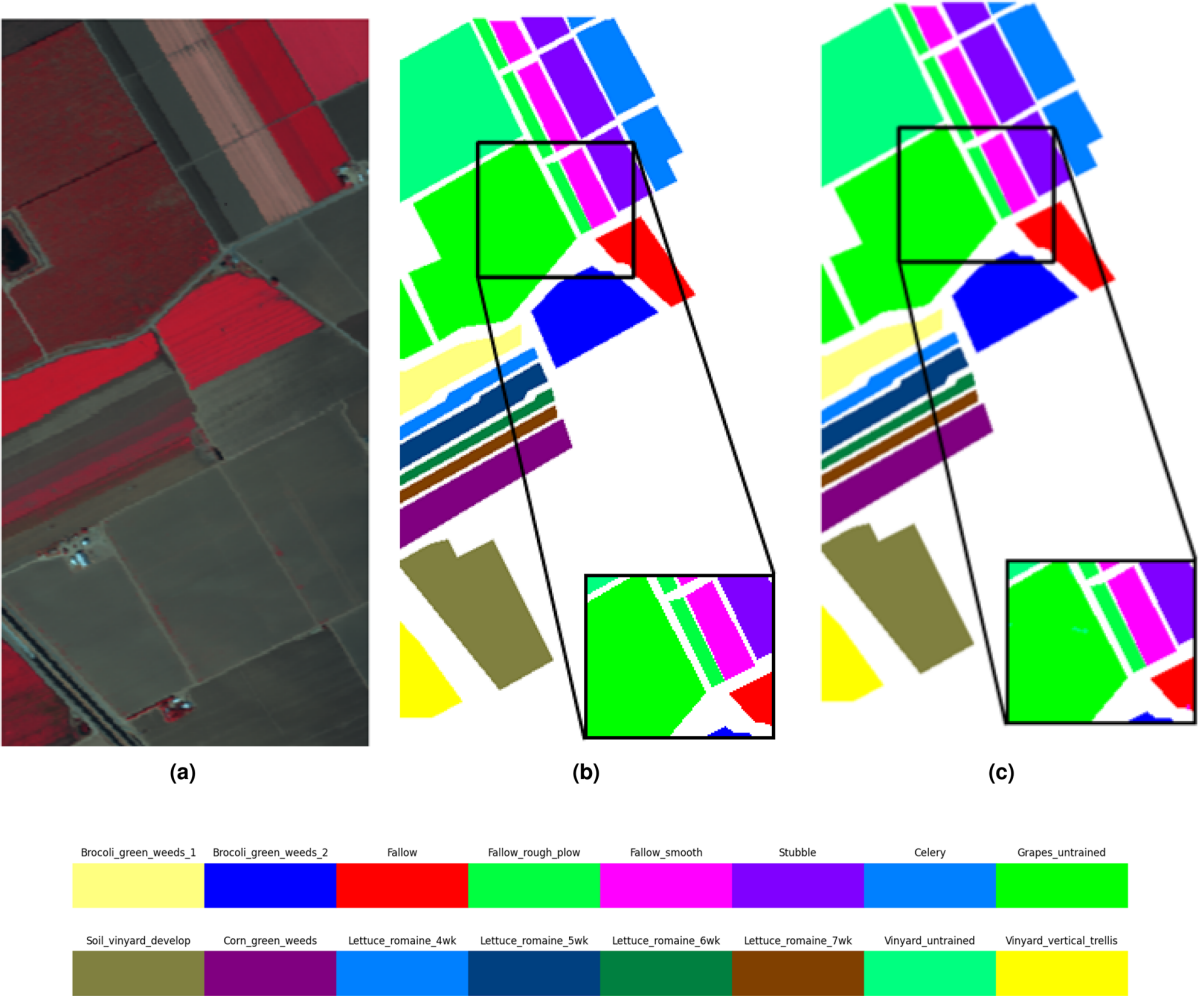
Classification results of on the Salinas Scene dataset (**a**) original HSI (**b**) ground truth (**c**) proposed method.

In addition to the above quantitative metrics, classification maps in the proposed method have been produced, as shown in Figs. 2, 3 and 4. Compared with ground truth, the proposed method obtains more accurate classification results, which further proves the effectiveness of the proposed method in the classification of hyperspectral data.

Figure 2 illustrates the classification results obtained using the DiffSpectralNet and the comparison algorithms on the Indian Pines dataset. The map highlights that the algorithm proposed in this study exhibits classification performance that closely resembles the actual terrain map of the Indian Pines dataset. The misclassification of terrain pixels is observed to be relatively minimal, resulting in a smoother overall effect. Notably, the algorithm demonstrates superior performance in classifying Grass-pasture-mowed, Oats, Wheat, and Woods features.

Moving forward, Fig. 3 provides a visual representation of the classification performance of the proposed model on the Pavia University dataset. The algorithm exhibited fewer misclassifications in the dataset, resulting in a smoother overall effect. Notably, in the classification of the Meadows, Metal sheets, and Bare soil features, the performance of the proposed algorithm is superior. This observation highlights the capability of the DiffSpectralNet to extract spectral and spatial information more comprehensively with the usage of the diffusion model.

Figure 4 presents the classification effect maps of the proposed model and the comparison algorithms on the Salinas Scene dataset. By observing the classification effect map of the model, it can be concluded that in the Brocoli_green_weeds_1, Brocoli_green_weeds_2, Fallow, Soil_vinyard_develop, Lettuce_romaine_4wk, Lettuce_romaine_5wk, Lettuce_romaine_6wk and Vinyard_vertical_trellis regions, there are fewer misclassified pixels of ground features compared with the comparison algorithms, resulting in a smoother overall effect map. This demonstrates that the DiffSpectralNet proposed in this paper can effectively reveal the intrinsic features hidden behind a HSI by learning low and high-level features.

For a comprehensive examination of the detailed performance metrics of each class for all three datasets, readers are directed to the Supplementary materials provided. In supplementary sections, we thoroughly compare our classification performance across various classes against a range of state-of-the-art methodologies to demonstrate the stability and reliability of our approach.

## Discussion

In this section, we explore further experiments and discussions on the following three aspects to explore the optimal classification performance and the application of the proposed model in practical remote sensing classification. First, we conduct experiments to discuss how to extract features from the pre-trained diffusion model to achieve optimal performance at various Timestamp and Feature index values. Second, we analyse the impact of the number of training samples directly affecting the network’s performance. Finally, we examine the influence of the quantity of PCA components on the spectral information in HSI datasets.

**Figure 5:**
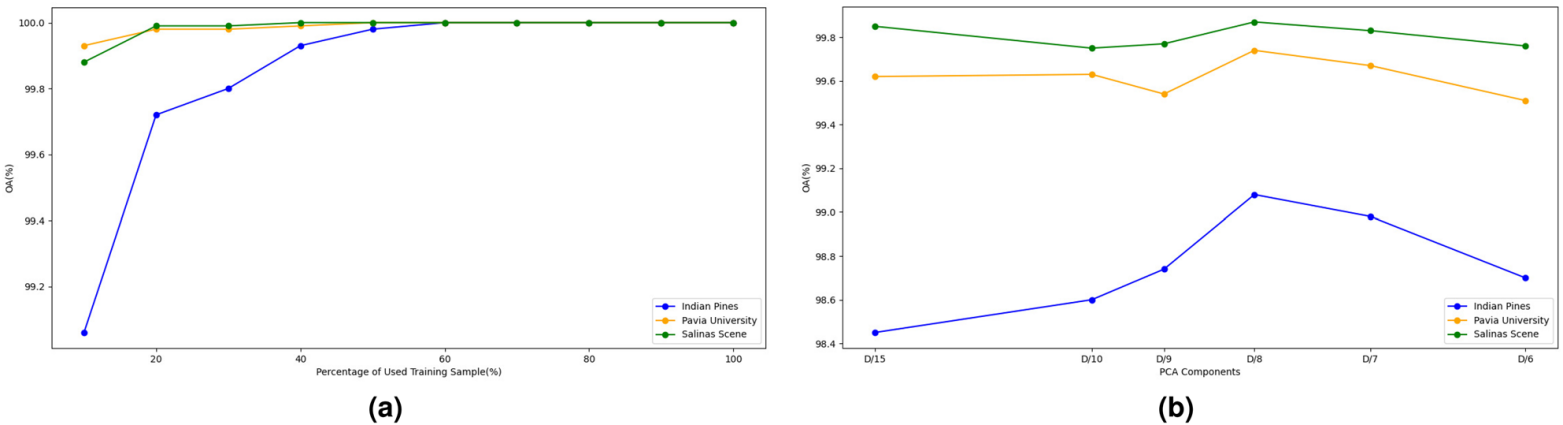
Classification accuracy (OA) achieved by the proposed DiffSpectralNet with (**a**) varying percentages of training samples (**b**) different PCA components on three benchmark datasets.

- Sensitivity analysis of Timestamps and Feature index: In order to analyse the features obtained from the diffusion pre-trained model, we have conducted classification experiments on various Timestamp and Feature index values and then recorded the change in the classification performance. Using the DDPM, we monitored classification efficacy alterations when Timestamp and Feature Index varied, and the optimal combination of Timestamp and Feature Index is essential to ensure accurate outcomes. Table 3 showcases the performance is sensative to Timestep and FeatureIndex. For the Indian Pines and Pavia University datasets, there is a certain correlation between Timestamp and FeatureIndex. When considering the Timestamp dimension, a decreasing trend in classification performance is observed when using features with larger Timestamps, and the optimal performance generally occurs in smaller Timestamp groups. Considering the FeatureIndex dimension, both datasets (Indian Pines and Pavia University) performed better at FeatureIndex 1 than at FeatureIndex 0 and 2. For Salinas Scene, there are some fluctuations in classification performance for different Timestamp and FeatureIndex values but no significant changes.
- Percentage of training samples: It is common knowledge that the number of training samples directly affects the performance of the network. To verify this with the proposed DiffSpectralNet, We evaluated the training dataset using random proportions ranging from 10 to 100% with increments of $10\%$, and depict the comparative results in Fig. 5a. As expected, the classification accuracy gradually improves with an increase in training samples. It is worth noting that OA tends to be stable when the percentage of training samples is greater than $50\%$. However, when the percentage of training samples in the Indian Pines dataset is less than $50\%$, the performance is unsatisfactory may be due to the insufficient number of samples for a proper training. Therefore, it is reasonable to extrapolate that DiffSpectralNet is reliable and stable for this task.
- Effect of PCA components on diffusion feature: We investigate the impact of the number of PCA components on the compressed spectral data. The data retain more spectral details with more PCA components but at the cost of increased computational demand and redundancy. The number of diffusion features varies across datasets, influencing the range of PCA components, which varies from D/6 to D/15, where D represents the diffusion features in a dataset. The results in Fig. 5b suggest optimal performance with D/8 PCA components.

**Table 3:** The performance of different layer indices and timestamps in the Indian Pines, Pavia University, and Salinas Scene.

FeatureIndex	Timestamp	Indian Pines			Pavia University			Salinas Scene		
		OA (%)	AA (%)	$\kappa$	OA (%)	AA (%)	$\kappa$	OA (%)	AA (%)	$\kappa$
0	5	98.47	95.37	0.9826	98.94	97.93	0.9860	99.74	99.73	0.9971
	10	98.41	96.40	0.9818	99.15	98.68	0.9887	**99.87**	**99.82**	0.9985
	100	97.92	96.85	0.9762	99.03	98.27	0.9871	99.71	99.67	0.9967
	200	97.62	94.45	0.9728	98.63	97.91	0.9818	98.63	97.91	0.9818
	400	98.15	96.38	0.9789	92.86	89.98	0.9053	98.29	97.74	0.9809
1	5	**99.06**	**98.00**	**0.9893**	**99.74**	99.16	**0.9965**	99.83	99.76	0.9981
	10	98.34	96.20	0.9811	99.63	99.09	0.9951	99.76	99.73	0.9973
	100	98.40	96.30	0.9817	99.54	**99.18**	0.9939	**99.87**	99.81	**0.9986**
	200	98.45	97.48	0.9823	98.79	97.53	0.9839	98.45	97.48	0.9823
	400	98.29	96.35	0.9805	92.61	88.75	0.9015	98.06	97.70	0.9784
2	5	98.59	95.17	0.9839	98.52	97.07	0.9803	99.26	99.32	0.9917
	10	98.82	94.99	0.9865	97.32	95.29	0.9644	98.95	99.00	0.9883
	100	98.01	96.05	0.9773	95.19	91.13	0.9361	98.04	97.98	0.9782
	200	96.37	93.26	0.9587	93.54	90.25	0.9139	96.37	93.26	0.9587
	400	95.71	92.52	0.9510	86.66	81.28	0.8202	91.84	88.39	0.9089

Significant values are in **bold**.

## Methods

In this section, we describe a novel method called DiffSpectralNet that consists of two stages: an unsupervised diffusion process and a supervised classification. The unsupervised diffusion process is derived from the DDPM with the purpose to learn spectral–spatial representations effectively. In this process, we extract plenty of spectral–spatial features from various time steps *t* during the reverse diffusion process of DDPM to capture the characteristics of different objects in HSI data. Finally, these features are inputted into the supervised classification model for classification.

**Figure 6:**
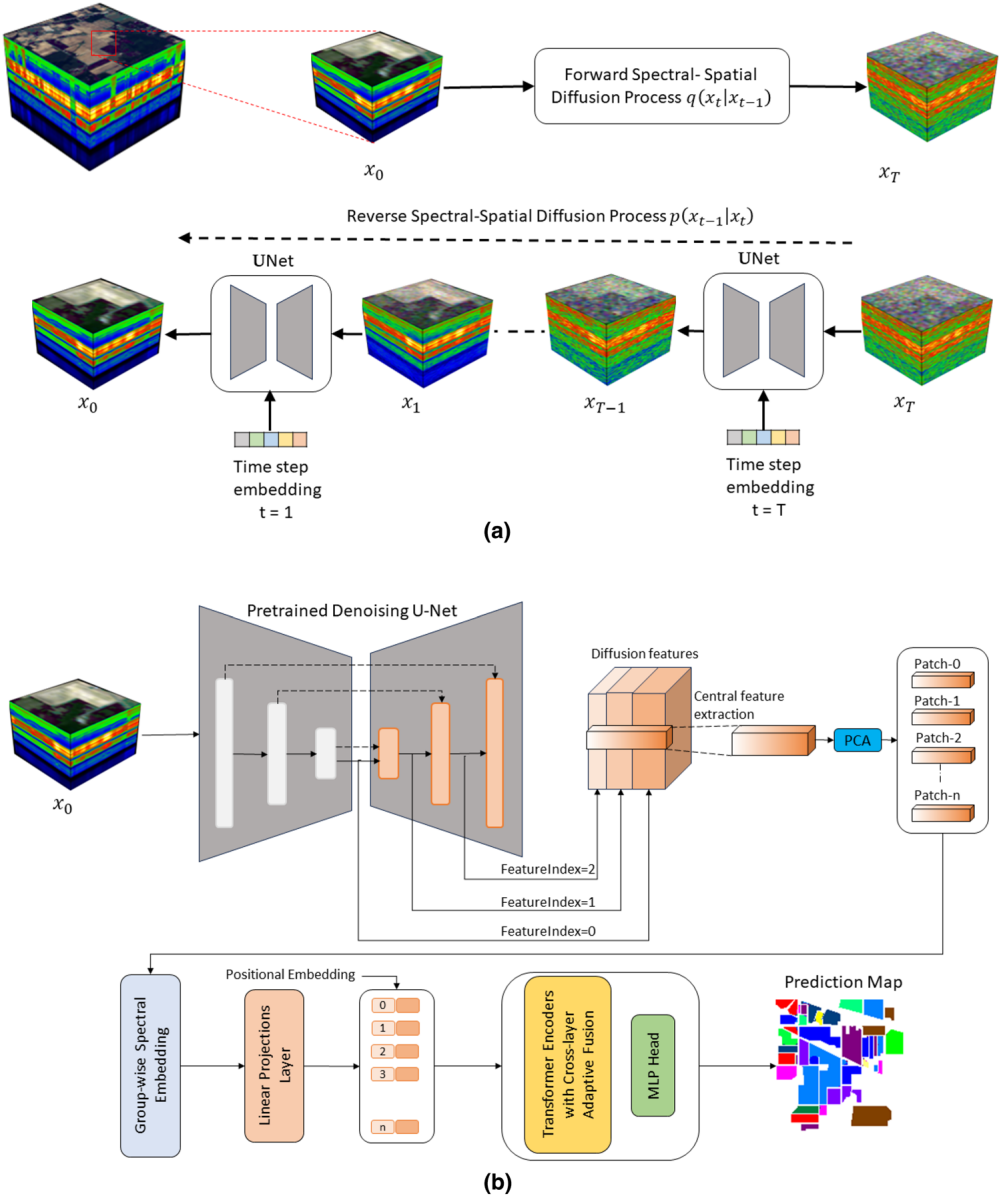
Overview of our proposed DiffSpectralNet (**a**) unsupervised spectral–spatial feature learning network. $x_0$ and $x_T$ represent HSI patches of timestep $0$ and timestep $T$. $q(x_t \mid x_{t-1})$ and $p(x_{t-1} \mid x_{t})$ represent forward and reverse spectral–spatial diffusion processes, respectively. (**b**) Supervised classification $(1)$ extracting hierarchical features from the pretrained denoising U-Net decoder in terms of different timestep t. $(2)$ Using the patch-wise feature vectors to train an cross-layer transformer for HSI classification.

### Diffusion-based unsupervised spectral–spatial feature learning

In order to capture complex spectral–spatial relations and label-agnostic information of HSI data effectively, the first step of our proposed approach is to train a diffusion model in an unsupervised manner, as shown in Fig. 6a. We introduce the detailed formulation of our unsupervised feature learning procedure, which involves diffusion-based forward and backward processes with the HSI data.

- Forward diffusion process: DDPM represents a category of models based on likelihood estimations. In the forward process, Gaussian noise is added to the original training data. In our proposed model, we aim to learn spectral–spatial features effectively in an unsupervised manner. We start by training our DDPM using unlabeled patches randomly cropped from the HSI dataset. To prepare the data for training, the data is pre-processed by patch cropping operation. Next, patches are randomly sampled from HSI for DDPM training. Formally, given an unlabeled patch $x_0 \in \mathbb {R}^{P \times P \times B}$, where *P* denote the height and width of patch $x_0$, *B* represents the number of spectral channels, respectively. During the forward diffusion process, Gaussian noise is gradually added to the HSI patch according to the variance schedule $\{\beta _t\}_{t=0}^{T}$ in the diffusion process where *T* is the total number of the timestep. The process follows the Markov chain^33^ process:
  1 $$\begin{aligned} q(x_t|x_{t-1}) = \mathscr {N}\left( \sqrt{(1 - \beta _t)}x_{t-1}, {\beta _t}{} \textit{I}\right) \end{aligned}$$
  where $\mathscr {N}$ is a Gaussian distribution. The above formulation leads to the probability distribution of the HSI at a given time $\textit{t} + 1$ is obtained by its state at time *t*. During the first diffusion, the spectral–spatial instance with noise is expressed as follows:
  2 $$\begin{aligned} x_{1} = \sqrt{\alpha _{1}}x_{0} + \sqrt{1 - \alpha _{1}}\varepsilon \end{aligned}$$
  At the $\textit{t}_{th}$ step, the spectral–spatial instance incorporated with noise is expressed as follows:
  3 $$\begin{aligned} x_t = \sqrt{\overline{\alpha }_t} x_0 + \sqrt{1 - \overline{\alpha }_t} \varepsilon , \quad \varepsilon \sim \mathscr {N}(0, \textit{I}) \end{aligned}$$
  where $\alpha _t$ = $1 - \beta _t$ and, $\overline{\alpha }_t$ represents the product of $\alpha _1$ to $\alpha _t$. Given these inputs, the hyperspectral instance at timestep *t* can be straightforwardly produced by Eq. (3).
- Reverse diffusion process: In the reverse diffusion process, a spectral–spatial **U**-Net^41^ denoising network is employed is trained to predict the noise added on $x_{t-1}$, taking noisy patch $x_t$ and timestep *t* as inputs. And $x_{t-1}$ is calculated by subtracting the predicted noise from $x_t$. DDPM uses a Markov chain process to remove the noisy sample $x_T$ to $x_0$ step by step. Under large *T* and small $\beta _t$, the probability of reverse transitions is approximated as a Gaussian distribution and is predicted by a **U**-Net as follows:
  4 $$\begin{aligned} p_{\theta }(x_{t-1}|x_{t}) = \mathscr {N} \left( x_{t-1}; \mu _{\theta }(x_{t}, t), \sigma _{\theta }(x_{t}, t) \right) \end{aligned}$$
  where the reverse process can be re-parameterized by estimating $\mu _{\theta }(x_{t}, t)$ and $\sigma _{\theta }(x_{t}, t)$. $\sigma _{\theta }(x_{t}, t)$ is set to $\sigma _t^2 I$, where $\sigma _t^2$ is not learned. To obtain the mean of the conditional distribution $p_{\theta }(x_{t-1}|x_{t})$, we need to train the network to predict the added noise. The mean of $\mu _{\theta }(x_{t}, t)$ is derived as follows:
  5 $$\begin{aligned} \mu _{\theta }(x_{t}, t) = \frac{1}{\sqrt{\alpha _t}} \left( x_{t} - \frac{1 - \alpha _t}{\sqrt{1 - \alpha _t}} \varepsilon _{\theta }(x_{t}, t) \right) \end{aligned}$$
  where $\varepsilon _{\theta }(\cdot , \cdot )$ denote the spectral–spatial denoising network whose input is the timestep *t* and the noisy hyperspectral instance $x_t$ at timestep *t*. The denoising network takes in the noisy hyperspectral instance along with the timestep to produce the predicted noise. The **U**-Net denoising model $\varepsilon _{\theta }(x_{t}, t)$ is optimised by minimising the loss function of the spectral–spatial diffusion process can be expressed as follows:
  6 $$\begin{aligned} \mathscr {L}(\theta ) = \mathbb {E}_{t, x_0, \varepsilon } \left[ \left( \varepsilon - \varepsilon _{\theta } \left( \sqrt{\overline{\alpha _t}} x_0 + \sqrt{1 - \overline{\alpha _t}} \varepsilon , t \right) \right) ^2 \right] \end{aligned}$$

### Supervised classification using spectral–spatial diffusion feature

After training the network using unsupervised spectral–spatial methods, we start extracting useful diffusion features from the pre-trained DDPM. Next, we employ a transformer-based classifier for classification.

During the feature extraction step, we utilize the **U**-Net denoising network to extract a spectral–spatial timestep-wise feature. The pre-training of DDPM enables it to capture rich and divers information from the input data during the reverse process. As a result, we extract features from the intermediate hierarchies of DDPM at various timesteps to create robust representations that encapsulate the salient features of the input HSI. The parameters of the pre-trained DDPM remain constant, as shown in Fig. 6b. We gradually add Gaussian noise to the input patch $x_0 \in \mathbb {R}^{P \times P }$ through the diffusion process. For a noisy input patch $x_t$ at timestep $t$, the noisy version $x_t$ can be directly determined using Eq. (3). Subsequently, $x_t$ is fed into the pre-trained spectral–spatial denoising **U**-Net to derive hierarchical features from the **U**-Net decoder. Diffusion features from various decoder layers are collectively upsampled to $P \times P$ and then merged to form the feature $f_t$ in $\mathbb {R}^{P \times P \times L}$ at timestep $t$, where $P$ represents the height and width of the patch and $L$ denotes the feature channel. For each feature $f_{ti} \in \mathbb {R}^{P \times P \times L}$, we retain only the vector associated with the center pixel, indexed as $C_i \in \mathbb {R}^{p \times p \times L}$. This approach significantly reduces the computational cost due to a decrease in parameters. We input the extracted diffusion features $(C(f_{ti})$ patch-wise to learn group-wise spectral embeddings. By proposing to learn group-wise spectral embeddings, we aim to precisely identify and classify the diverse features based on their distinct spectral properties. The group-wise spectral embedding features use a linear projection layer for mapping features to a token sequence for the transformer. Positional embedding is added to the input token sequence before feeding it to the transformer. This provides the transformer with information about the relative positions of the patches. Therefore, the abundant features contain diverse and multi-level information of the input HSI data, which we use for classification.

After mapping the patch representation, a network is needed to predict the classification label. Transformer-based classifiers are trained based on the inspiration from^24^, as shown in Fig. 6b. The classification module combines the CNN and transformer structures to form an effective classifier. These classifiers take positionally embedded feature patches as inputs and use an MLP head to predict the final classification scores. Inspired by the success of skip connection in U-Net^42^, and ResNet^16^ for image segmentation and recognition, respectively. A cross-layer skip connection is introduced in the classifier to minimise the possibility of losing valuable information in the layer-wise propagation process and enhance the information transitivity between layers. The classifier model utilises skip connection, multi-head attention mechanisms, feed-forward neural networks to spectral–spatial feature mapping, and a transformer structure for deep feature extraction, resulting in outstanding classification performance.

## Conclusion

HSI contains rich spectral–spatial information and complex relations, which are critical for classification tasks. The proposed method provides a unique viewpoint for the spectral–spatial diffusion process, which is capable of modeling complex relationships for understanding inputs and learning both high-level and low-level features. In conclusion, most current methods for HSI classification rely on CNN or Transformer models, which may not efficiently extract patterns and information. In contrast, our proposed method, employing the diffusion model, effectively and efficiently learns discriminative spectral–spatial features. This approach allows us to explore and utilise the spatial–spectral neighborhood structure of hyperspectral data, resulting in the effective extraction of deep features. Instead of processing on a pixel-by-pixel basis, the diffusion features are introduced in patches to improve the ability to capture details for more accurate classification. We employed a transformer-based model with a cross-layer skip connection, which reduces the possibility of losing valuable information in the layer-wise propagation process. We demonstrated the superiority of our proposed DiffSpectralNet approach by achieving state-of-the-art results in HSI classification based on quantitative trials conducted on three HSI datasets. In future studies, we aim to validate and enhance the performance of our proposed model on additional hyperspectral datasets across various domains, such as the medical field. Our model can be generalised and shows promise in HSI classification due to its ability to capture complex relationships between bands.

### Supplementary Information

Supplementary Information.

## Data Availability

The datasets analysed during the current study are available in the Grupo de Inteligencia Computacional (GIC) Hyperspectral Remote Sensing Scenes. Supplementary information is available on the online version of the paper which shows the detailed information of these three datasets.
