# Supplementary material for: Unveiling the potential of diffusion model-based framework with transformer for hyperspectral image classification
Source: Sci Rep. 2024 Apr 10;14:8438. doi: 10.1038/s41598-024-58125-4 (PMC11006679; doi:10.1038/s41598-024-58125-4)
Supplement: Supplementary file 1 — Supplementary Information. [file 41598_2024_58125_MOESM1_ESM.pdf]

Unveiling the Potential of Diffusion Model-Based Framework with Transformer for Hyperspectral Image Classification - Supplementary Material

Neetu Sigger[1], Quoc-Tuan Vien[2], Sinh V. Nguyen[3], Gianluca Tozzi[4], and Tuan T. Nguyen[5,*]

[1]The University of Buckingham, School of Computing, Buckingham, MK181EG, United Kingdom
[2]Middlesex University, Faculty of Science and Technology
[3]International University–Vietnam National University of HCMC, School of Computer Science and Engineering, VN
[4]University of Greenwich, School of Engineering, Chatham Maritime, ME44TB, United Kingdom
[5]University of Greenwich, School of Computing & Mathematical Sciences, London, SE109LS, United Kingdom
[*]tuan.nguyen@greenwich.ac.uk

Quantitative Results and Analysis:

We evaluate the performance using three prominent metrics: Overall Accuracy (OA), Average Accuracy (AA), and Kappa Coefficient (κ). To demonstrate the effectiveness of our proposed DiffSpectralNet, we compare our classification performance for each class with various state-of-the-art approaches. The following methods were chosen: DMVL + SVM[1], 3DCAE[2], GSSCRC[3], SS1DSwin[4], SpectralFormer[5] and SpectralDiff[6]. Note that, due to unavailability of source code for certain methods, we directly used the reported results of each method from their respective papers, except for the Salinas Scene results of SpectralFormer, which were not available. Instead, we conducted tests on the same train-test samples used in our research. Both the CNN-based and transformer-based methods achieved very good classification results.

Based on the data presented in Table S1, it is evident that the DiffSpectralNet proposed in this study shows better classification accuracy for most ground objects when compared to other classification methods. Table S1 illustrates the classification results obtained using the DiffSpectralNet and the comparison algorithms on the Indian Pines dataset. Notably, the model demonstrates superior performance in the classification of class 7, 9, 13, and 14 features with 100% accuracy.

Table S1. Classification accuracy for the proposed and compared HSI classification methods on the Indian Pines dataset (the best accuracy in each row is shown in bold).

Class	**Sample Size**		**Classification Methods**						
	Training	Test	DMVL+SVM	3DCAE	GSSCRC	SS1DSwin	SpectralFormer	SpectralDiff	Ours
1	5	41	86.96	90.48	**100.00**	92.31	79.49	**100.00**	87.80
2	143	1285	67.51	92.49	90.97	83.89	70.52	85.47	**98.67**
3	83	747	72.53	90.32	88.07	88.65	81.89	94.12	**98.13**
4	24	213	**99.58**	86.90	84.39	98.91	91.30	**100.00**	97.65
5	48	435	68.12	94.25	95.65	95.30	95.53	96.02	**99.54**
6	73	657	76.16	97.07	98.77	97.42	85.51	**99.85**	99.54
7	3	25	**100.00**	91.26	**100.00**	**100.00**	**100.00**	**100.00**	**100.00**
8	48	430	**100.00**	97.79	99.79	**100.00**	99.32	**100.00**	99.77
9	2	18	**100.00**	75.91	**100.00**	87.04	**100.00**	**100.00**	**100.00**
10	97	875	83.64	87.34	90.53	87.04	**100.00**	84.50	99.89
11	245	2210	71.41	90.24	90.24	83.13	79.49	91.95	**99.14**
12	59	534	49.07	95.76	94.94	85.28	**100.00**	95.56	98.69
13	20	185	98.05	97.49	99.51	**100.00**	**100.00**	**100.00**	
14	126	1139	99.05	96.03	97.63	98.63	93.17	97.08	**100.00**
15	39	347	87.56	90.48	79.53	**100.00**	81.81	**100.00**	98.85
16	9	84	**100.00**	98.82	**100.00**	**100.00**	73.76	98.41	90.48
	OA (%)		78.01	92.354	91.33	89.66	81.76	93.14	**99.06**
	AA (%)		84.98	92.04	93.81	94.13	87.81	96.43	**98.00**
	κ		0.7531	-	0.9013	0.8819	0.7919	92.17	**0.9893**

Next, the experiment was conducted on the Pavia University dataset. The detailed classification results of the proposed model and other algorithms for different features are presented in Table S2. The model showed fewer misclassifications in the dataset, resulting in a smoother overall effect; the performance of the proposed algorithm surpassed that of other algorithms on 7 classes.

Table S2. Classification accuracy for the proposed and compared HSI classification methods on the Pavia University dataset (the best accuracy in each row is shown in bold).

Class	**Sample Size**		**Classification Methods**						
	Training	Test	DMVL+SVM	3DCAE	GSSCRC	SS1DSwin	SpectralFormer	SpectralDiff	Ours
1	332	6299	57.80	90.48	96.20	89.99	82.73	86.15	**99.98**
2	932	17717	98.32	92.49	98.44	94.81	94.03	98.39	**100.00**
3	105	1994	84.37	90.37	83.66	84.85	73.66	96.56	**99.95**
4	153	2911	56.01	86.90	96.21	91.93	93.75	76.40	**98.56**
5	67	1278	**100.00**	94.25	98.85	99.64	99.28	99.54	**100.00**
6	251	4778	**100.00**	97.07	99.92	95.14	90.75	98.30	**100.00**
7	67	1263	**99.85**	91.26	94.82	88.07	87.56	99.07	98.50
8	184	3498	97.23	97.79	91.91	91.41	95.81	98.98	**99.89**
9	47	900	27.35	75.91	**99.89**	91.45	94.21	91.16	95.56
	OA (%)		86.96	87.34	95.77	93.04	91.07	94.77	**99.74**
	AA (%)		80.10	90.24	94.13	91.92	90.20	93.84	**99.18**
	κ		0.8246	-	0.9438	0.9068	0.8805	93.06	**0.9965**

Table S3 presents the classification results on the proposed algorithm and the comparison algorithms on the Salinas Scene dataset. By observing the classification effect of each model, it can be concluded that the model demonstrates superior performance in the classification of 8 classes of features with 100% accuracy.

Table S3. Classification accuracy for the proposed and compared HSI classification methods on the Salinas Scene dataset (the best accuracy in each row is shown in bold).

Class	Sample Size		Classification Methods						
	Training	Test	DMVL+SVM	3DCAE	GSSCRC	SS1DSwin	SpectralFormer	SpectralDiff	Ours
1	100	1909	95.92	**100.00**	**100.00**	**100.00**	97.64	**100.00**	**100.00**
2	186	3540	**100.00**	99.29	**100.00**	99.97	99.97	**100.00**	**100.00**
3	98	1878	**100.00**	97.13	99.80	99.79	99.57	**100.00**	**100.00**
4	69	1325	**100.00**	97.91	99.92	96.99	98.33	**100.00**	99.85
5	133	2545	94.73	98.26	99.10	98.77	99.76	98.03	**99.84**
6	197	3762	90.96	99.98	99.72	**100.00**	**100.00**	**100.00**	99.89
7	178	3401	98.60	99.64	99.86	**99.94**	99.35	99.23	99.79
8	563	10708	91.47	91.58	89.76	87.67	92.86	96.97	**99.74**
9	310	5893	99.92	99.28	**100.00**	99.90	99.21	**100.00**	**100.00**
10	163	3115	80.63	96.65	98.32	95.76	98.20	98.61	**99.94**
11	53	1015	99.25	97.74	98.65	98.00	96.84	99.80	**100.00**
12	96	1831	94.91	98.84	**100.00**	**100.00**	99.78	99.94	**100.00**
13	45	871	72.49	99.26	99.13	**100.00**	99.08	**100.00**	**100.00**
14	53	1017	98.97	97.49	95.70	99.00	98.12	**100.00**	98.13
15	363	6905	95.56	87.85	77.43	89.27	87.51	98.82	**99.99**
16	90	1717	**100.00**	98.34	99.45	99.37	98.77	**100.00**	**100.00**
	OA (%)		94.60	95.81	95.62	95.45	96.27	98.97	**99.87**
	AA (%)		94.59	97.45	97.30	97.78	97.82	99.46	**99.82**
	κ		0.9400	-	0.9384	0.9493	95.85	0.9885	**0.9986**

References

1. Liu, B. *et al.* Deep multiview learning for hyperspectral image classification. *IEEE Transactions on Geosci. Remote. Sens.* **59**, 7758–7772, DOI: 10.1109/TGRS.2020.3034133 (2021).
2. Mei, S. *et al.* Unsupervised spatial–spectral feature learning by 3d convolutional autoencoder for hyperspectral classification. *IEEE Transactions on Geosci. Remote. Sens.* **57**, 6808–6820, DOI: 10.1109/TGRS.2019.2908756 (2019).
3. Zheng, G. *et al.* Hyperspectral image classification using geodesic spatial spectral collaborative representation. *Electronics* **12**, DOI: 10.3390/electronics12183777 (2023).
4. Xu, Y. *et al.* Spatial–spectral 1dswin transformer with groupwise feature tokenization for hyperspectral image classification. *IEEE Transactions on Geosci. Remote. Sens.* **61**, 1–16, DOI: 10.1109/TGRS.2023.3294424 (2023).
5. Hong, D. *et al.* Spectralformer: Rethinking hyperspectral image classification with transformers. *IEEE Transactions on Geosci. Remote. Sens.* **60**, 1–15, DOI: 10.1109/TGRS.2021.3130716 (2022).

6. Chen, N., Yue, J., Fang, L. & Xia, S. Spectraldiff: A generative framework for hyperspectral image classification with diffusion models. *IEEE Transactions on Geosci. Remote. Sens.* **61**, 1–16, DOI: 10.1109/tgrs.2023.3310023 (2023).
